# Staged correction trajectory with hexapod external fixator for the satisfactory reduction of long bone shaft fracture

**DOI:** 10.1186/s12891-022-05172-5

**Published:** 2022-03-08

**Authors:** Yanshi Liu, Fei Wang, Kai Liu, Feiyu Cai, Xingpeng Zhang, Hong Li, Tao Zhang, Aihemaitijiang Yusufu

**Affiliations:** 1 Department of Trauma and Microreconstructive surgery, the First Affiliated Hospital of Xinjiang Medical University, Urumqi, Xinjiang, China; 2 Department of Orthopaedics, the First Affiliated Hospital of Xinjiang Medical University, Urumqi, Xinjiang, China; 3 Department of Orthopedics, Shanghai Pudong New Area People’s Hospital, Shanghai, China; 4 Department of Orthopedics, Zigong Fourth People’s Hospital, Zigong, Sichuan China; 5 Department of Orthopedics and Trauma, Tianjin Hospital, Tianjin, China

**Keywords:** Fracture reduction, Hexapod external fixator, Staged correction trajectory, Tibial shaft fractures

## Abstract

**Backgroud:**

When the reduction of long bone shaft fracture fragments is performed by a hexapod external fixator, the collision and interference between bony ends often results in an incomplete reduction and a time-consuming procedure. The purpose of this study was to present and determine the clinical effectiveness of staged correction trajectory with hexapod external fixator in the reduction of a long bone shaft fracture.

**Methods:**

A total of 57 patients with tibial shaft fractures treated by hexapod external fixator were retrospectively analyzed from June 2016 to February 2020. Thirty-one cases (Group I) underwent a conventional one-step reduction trajectory from June 2016 to July 2018. Starting in September 2018, the other twenty-six patients (Group II) underwent staged correction trajectory (three key points reduction trajectory of “distraction-derotation-reduction”). The demographic data, residual deformities before and after correction, number of repeated X-rays after the first postoperative X-ray, duration of deformity correction process, and external fixation time were analyzed. Johner-Wruhs criteria were used to evaluate the final clinical outcomes.

**Results:**

All the 57 patients achieved satisfactory fracture reduction and bone union. There were no significant differences between the two groups in demographic data, residual deformities before and after correction, external fixation time, and final clinical outcomes (*p* > 0.05). The average number of repeated X-rays after the first postoperative X-ray and mean duration of deformity correction process in Group II (1.3 times, 2.9 days) were all less than those in Group I (2.3 times, 5.1 days) (*p* < 0.05).

**Conclusion:**

Compared with the conventional one-step reduction trajectory, there is no differences in final clinical outcomes, but the staged correction trajectory provides less repeated X-rays and shorter reduction process duration.

## Background

Circular external fixators provide both stable three-dimensional fixation and a degree of axial micromotion, promoting bone healing process and regeneration [1–3]. Minimal soft tissue damage and possibility of early weight bearing give to expect a good final outcome in bone fractures external fixation, especially in high energy fractures accompanied by poor surrounding soft tissues [4–7].

Hexapod external fixation (HEF) systems, such as the Taylor spatial frame (TSF) and TrueLok-Hex (TL-Hex), are derived from the traditional Ilizarov circular external fixator [8]. The HEF consists of two full or partial rings connected by six telescopic struts at special universal joints, providing the frame with six degrees of freedom. In this frame, the position of one ring can be changed in all space dimensions relating to the other one, by adjusting each strut length. This feature allowes the device to be used in bone deformities correction too. Over time, the hexapod external fixator has become more and more popular both in trauma-control and in definitive treatment of high-energy fractures [4, 9–12]. Stable contact between bone and HEF is necessary in fracture reduction or deformity correction planning based on x-rays. But, despite this stable contact, a try of one-step reduction trajectory can result in malposition due to the contact collision between bone fragments. Thenafter there is the need for additional reduction procedures and new X-rays checks, exposing the patient to further radiation.

At our department, staged correction procedures called three key point trajectory of “distraction-derotation-reduction” were applied in fracture reduction process to resolve the contact collision between bone fragments. The purpose of this study was to present and to analyse the clinical significance of staged correction trajectory with hexapod external fixator in long bone shaft fracture reduction.

## Methods

This retrospective study included 57 patients with acute tibial shaft fractures treated by hexapod external fixator (Tianjin Xinzhong Medical Instrument Co., Ltd., Tianjin, China) at the department of Trauma and Microreconstructive surgery, the First Affiliated Hospital of Xinjiang Medical University, from June 2016 to February 2020, including 39 males and 18 females with a mean age of 41 years (range 19–65 years). The hexapod external fixation treatments were conducted due to trauma-control of high-energy complex fractures, fractures with poor surrounding soft tissues that were not suitable for internal fixation, and fractures that needed delayed soft tissue reconstruction. In any anatomical plane, postoperative deformities greater than 5° or 10 mm need to be corrected [13].

Thirty-one cases (Group I) underwent a conventional one-step reduction trajectory from June 2016 to July 2018. Starting in September 2018, the other twenty-six patients (Group II) underwent staged correction trajectory (three key points reduction trajectory of “distraction-derotation-reduction”). All the treating procedures were performed by the same medical team. The demographic data, residual deformities before and after correction, number of repeated X-rays after the first postoperative X-ray, duration of deformity correction process, and external fixation time in all cases were documented and analyzed. This study was approved by the Ethical Committee of our institution.

### Fracture reduction procedures

The residual deformities were evaluated by the immediately postoperative orthogonal anteroposterior (AP) and lateral X-ray, followed by the application of total residual program in the HEF system. Any deformities were corrected within 3 days by gradual strut adjustment according to the instrutions of computer analysis followed by oral analgesics were used in pain management. Repeated X-rays and computer analysis were performed until satisfactory reduction was achieved.

In Group I (one-step reduction trajectory), all deformity parameters (angulation and translation in the anteroposterior, lateral, and axial view) were inputted into the HEF system program at once, and the deformity correction process was then performed according to the computer instructions.

**Fig. 1:**
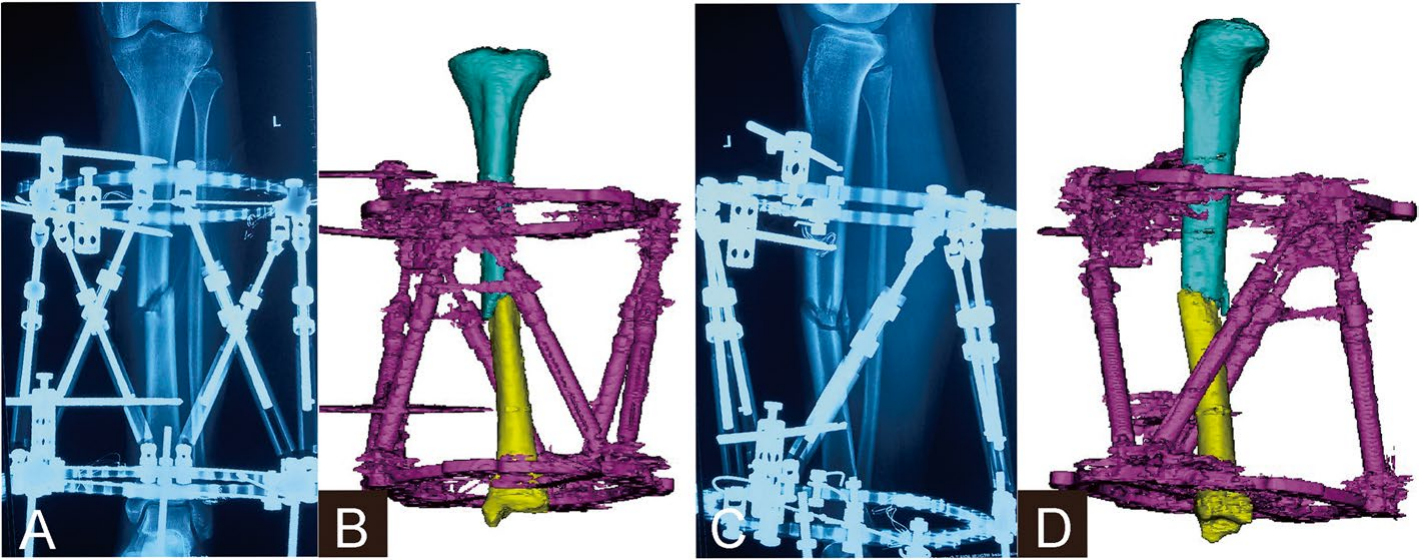
A 41-year-old man with traumatic multidimensional displacement in tibia treated by the hexapod external fixator, and underwent staged correction trajectory. **a** and **b** Immediate postoperative X-rays and three-dimensional reconstruction in the AP view. **c** and **d** Immediate postoperative X-rays and three-dimensional reconstruction in the lateral view

**Fig. 2:**
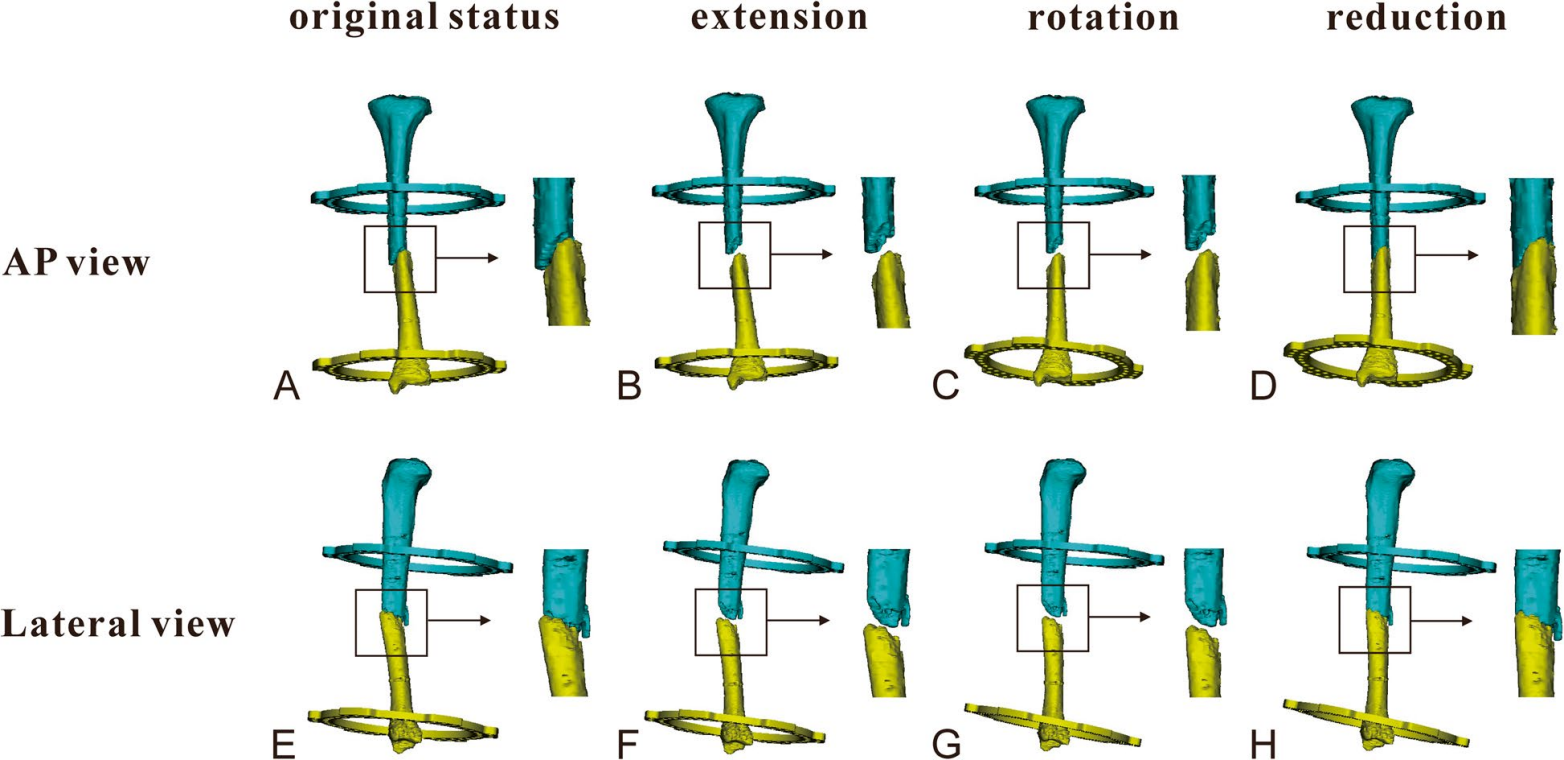
Scheme of the staged correction trajectory for the case from Fig. 1. **a** and **e** Primary position. **b** and **f** “Distraction” key point, the distal fragment was moved at an appropriate distance from the proximal fragment, providing sufficient space for the fracture reduction maneuver without bone fragments collision. **c** and **g** “Derotation” key point, the distal fragment was moved and rotated in multiple planes to correct the displacement. **d** and **h** “Reduction” key point, the ends of two bone fragments were docked and the final fracture reduction was achieved

As for Group II (staged correction trajectory), three key points reduction trajectory was performed (Figs. 1-2). A translation parameter in the axial view (within 10 mm according to our experience) was inputted into the HEF system program firstly to determine the lengthening of the given case, while the other five deformity parameters were set to zero. The first step of fracture reduction was performed according to the electronic prescription of the “distraction” key point (Fig. 2b and f). Subsequently, the five deformity parameters according to the postoperative X-rays (including angulation and translation in the AP and lateral view, angulation in the axial view) were inputted to determine rotation, while the translation parameter in the axial view was set to zero. The second step of fracture reduction was conducted depending on the electronic prescription of the “derotation” key point (Fig. 2c and g). Finally, a translation parameter in the axial view (original deformity combined with the “given” deformity in the first step) was inputted to determine the shortening, the other five deformity parameters were set to zero at the same time. The final fracture reduction was achieved using the electronic prescription of the “reduction” key point (Fig. 2d and h).

### Clinical effectiveness evaluation

The reduction effectiveness was evaluated by the translation and angulation in the AP and lateral view, according to the standard orthogonal X-rays after the final reduction. The residual deformities were assessed by the same observer who was experienced in musculoskeletal radiology analysis using CorelDRAW X7 software.

The patients were followed up monthly during the fracture healing time. The hexapod external fixation was terminated when X-rays confirmed sufficient union (corticalization in 3 of 4 cortices). All patients were followed up for a minimum of 12 months after the fixator removal. The final clinical outcomes were evaluated by the Johner-Wruhs criteria [14] at the last clinical exam.

### Statistical analysis

The SPSS 22.0 software was used for statistical analysis. Continuous variables were analyzed by Independent-samples T-test. Count variables were analyzed by the Chi-square or Fisher’s test. A statistically significant difference was set for *p* < 0.05.

## Results

All the 57 patients achieved satisfactory fracture reduction and bone union. The average follow-up after the hexapod external fixator removal was 17.9 months (12–26 months), and no case was lost. Superficial pin tract infection was the most common complication during the external fixation treatment as expected, and they were successfully managed by daily pin site care and oral antibiotics. In addition, no patient developed sequestrum requiring debridement. Furthermore, no wires or pins loosening, reduction loss, neurovascular injury, and refracture were observed. Activities of daily life without significant difficulty were performed in all patients at the last exam.

**Fig. 3:**
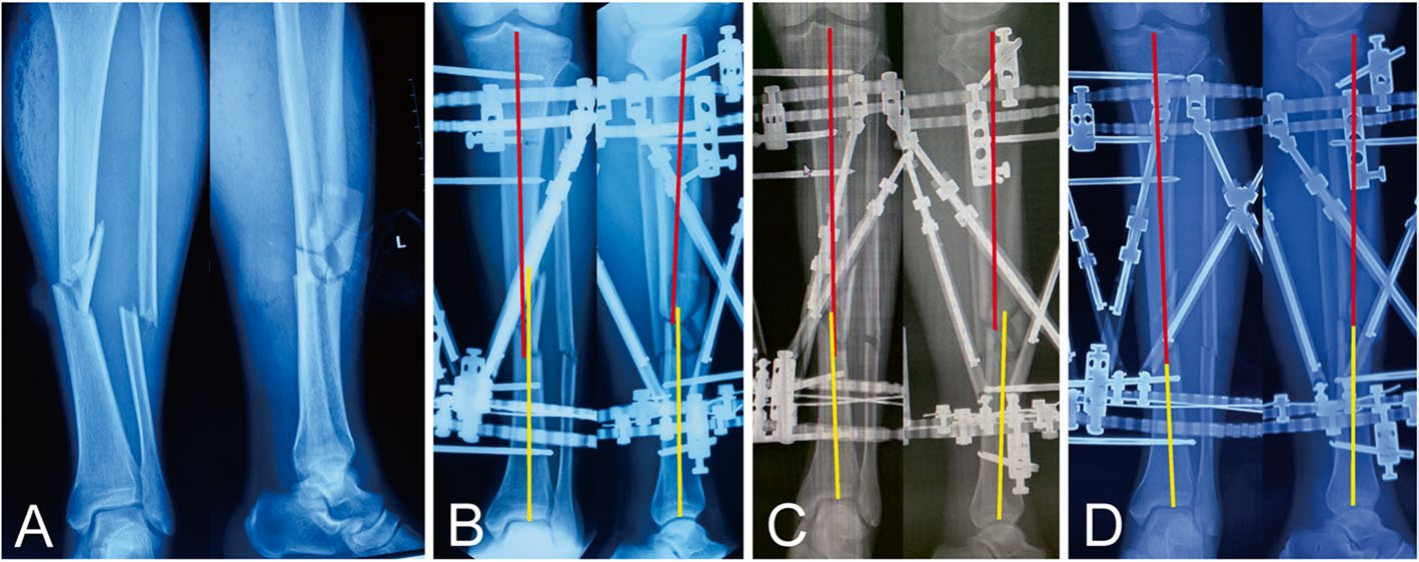
A 49-year-old man with traumatic multidimensional displacement in tibia treated by the hexapod external fixator, performing conventional one-step reduction trajectory, this patient underwent two repeated X-rays after the first postoperative X-ray, and the deformity correction process took 6 days. **a** Traumatic X-rays. **b** X-rays immediately after surgery. **c** X-rays after the first correction. **d** X-rays after the second correction

**Fig. 4:**
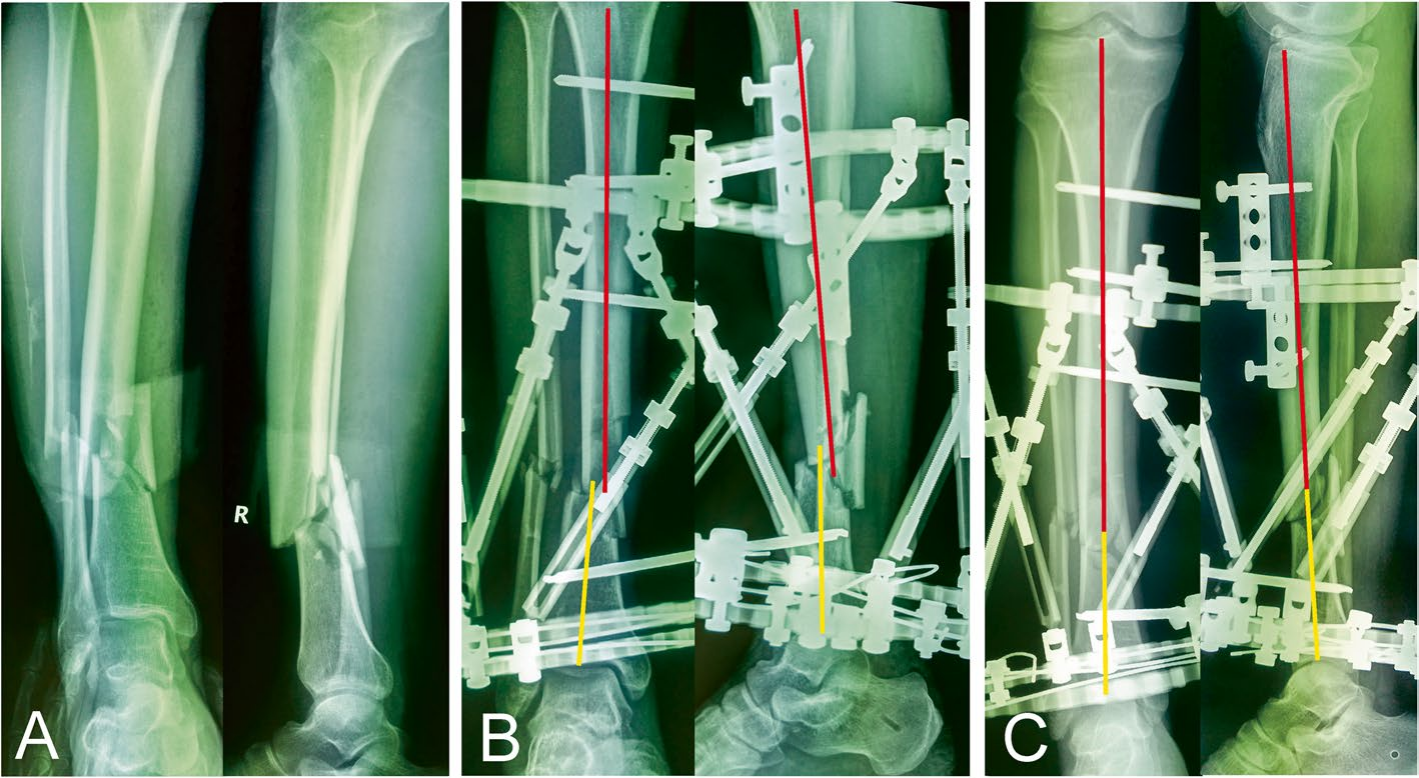
A 52-year-old man with traumatic multidimensional deformity in tibia treated by the hexapod external fixator, performing staged correction trajectory, this patient had one repeated X-ray exposure after the first postoperative X-ray and a three-day of deformity correction process. **a** Traumatic X-rays. **b** X-rays after surgery. **c** X-rays after the first correction

There were no significant differences between the two groups in demographic data, residual deformities before and after correction, and external fixation time (*p* > 0.05). The average number of repeated X-rays after the first postoperative X-ray and mean duration of deformity correction process in Group II (1.3 times, 2.9 days) were less than those in Group I (2.3 times, 5.1 days) (*p* < 0.05). Typical cases are presented in Figs. 3 and 4.

Based on the Johner-Wruhs criteria, in Group I, there were excellent in 23 cases, good in 6 cases, and moderate results in 2 cases. Excellent in 19 patients, good in 5, and moderate results in 2 were observed in Group II. There were no statistically significant differences between the two groups (*p* > 0.05). More details are shown in Tables 1 and 2.

**Table 1:** Demographic data of the two groups

	Group I	Group II	t/χ^2^	*p* value
Patients				
Male	21	18	0.015	0.904
Female	10	8		
Age (year)	40.6 ± 11.9	41.1 ± 9.9	−0.170	0.865
Injury mechanism				
Road traffic accident	20	18	1.380	0.531
Fall from height	4	5		
Crushing injury	7	3		
Open/closed fracture				
Open	22	16	0.566	0.575
Closed	9	10		
Gustilo classification of the open fractures				
Type I	3	1	–	0.876
Type II	4	3		
Type IIIA	13	9		
Type IIIB	2	3		
Type IIIC	0	0		
OTA classification of fractures				
A	11	6	1.251	0.599
B	16	17		
C	4	3		
Time elapsed since the injury to HEF installation (day)	3.3 ± 1.4	3.1 ± 1.1	0.332	0.741

**Table 2:** Clinical outcomes of the two groups

	Group I	Group II	t/χ^2^	*p* value
Residual deformities before correction				
T1(mm)	9.6 ± 5.6	8.5 ± 5.2	0.745	0.459
A1(°)	5.5 ± 2.5	5.1 ± 3.3	0.596	0.553
T2(mm)	8.3 ± 3.3	8.9 ± 5.1	−0.505	0.616
A2(°)	3.8 ± 1.9	4.4 ± 2.1	−1.204	0.234
Residual deformities after correction				
T1(mm)	1.8 ± 1.3	1.2 ± 1.1	1.748	0.086
A1(°)	0.7 ± 0.8	0.6 ± 0.8	0.720	0.475
T2(mm)	1.2 ± 1.1	0.9 ± 1.3	0.859	0.394
A2(°)	0.8 ± 0.9	0.7 ± 0.9	0.777	0.441
N (time)	2.3 ± 1.0	1.3 ± 0.5	4.572	*P* < 0.001
Duration of deformity correction (day)	5.1 ± 1.9	2.9 ± 1.1	4.914	*P* < 0.001
External fixation time (week)	27.9 ± 4.9	27.2 ± 1.9	0.543	0.589
Follow-up (month)	18.3 ± 3.7	17.5 ± 4.7	0.699	0.488
Johner-Wruhs criteria				
Excellent	23	19	0.212	1.000
Good	6	5		
Moderate	2	2		
Poor	0	0		

T1: Residual translation in the coronal plane

A1: Residual angulation in the coronal plane

T2: Residual translation in the sagittal plane

A2: Residual angulation in the sagittal plane

N: number of repeated radiographs after the first postoperative radiograph

## Discussion

Combined with the Ilizarov circular external fixator and the Chasles theorem of six-axis motion [15, 16], the hexapod external fixator has played an important role in orthopedic and reconstructive surgery due to the unique type of multiplanar spatial deformities correction [4, 7–10, 12, 17, 18]. Initially developed for gradual deformity correction process, the hexapod external fixator expanded its use in fracture and bone nonunion treatment [11, 12, 18–20].

Using the hexapod external fixator, high theoretical accuracies of 1/1000000 in. and 1/10000 degrees are extreme for clinical practice, but it is acceptable in real to approximate correction accuracies of 1 mm and 1° [15, 21]. Accurate X-ray analysis of deformity and mounting parameters are crucial for the success of hexapod external fixation treatment. Although lots of satisfactory clinical outcomes have been manifested in the HEF treatment, no technique is perfect in fact, as most parameter measurement techniques are subjective and depend on human factors. Malcorrection or insufficient correction can result from some subtle errors in the parameter definition. Lots of previously published methods have been described to improve parameter accuracy, including CT scans, intraoperative fluoroscopy, postoperative X-rays, and determination of the X-ray orthogonality [15, 22–30].

Abundant efforts have been described to obtain the standard orthogonal radiographs. Gantsoudes et al. [15] utilized equipment that is already available in a TSF treatment to obtain intraoperative orthogonal images, pointing out that their technique was quick, cheap, and easily reproducible. Ahrend et al. [28] had taken postoperative X-rays using a rotation rod, and the results suggested that the variability of rotation on X-rays was lower with the rotation rod, and more comparable X-rays could be obtained. Kanellopoulos et al. [24] described a noninvasive technique using a specially designed radiolucent frame to determine the reference ring precisely orthogonal in single exposures for each X-ray view. Deakin DE et al. [25] acquired perfectly aligned X-rays with the help of a frame-mounted spirit level. Wright et al. [27] described a silhouette technique to produce adequate orthogonal imaging. (shown in Table 3).

**Table 3:** Summary of literatures on perfecting orthogonality of radiographs

Method	Title	Author
Markers	Intraoperative measurement of mounting parameters for the Taylor Spatial Frame	Gantsoudes et al. [15]
Rotation rod	Improving the accuracy of patient positioning for long-leg radiographs using a Taylor Spatial Frame mounted rotation rod	Ahrend et al. [28]
Guide frame	A guide frame for the Taylor Spatial Frame	Kanellopoulos et al. [24]
Spirit level	A frame-mounted X-ray guide for the Taylor Spatial Frame	Deakin DE et al. [25]
Silhouette technique	The silhouette technique: improving post-operative radiographs for planning of correction with a hexapod external fixator	Wright et al. [27]
Guideline	Improving radiographic imaging for circular frames: the Cambridge experience.	Al-Uzri et al. [29]
Additional foot ring	Improving postoperative radiographs for the parameter measurement of hexapod external fixator using an additional foot ring	Liu et al. [30]

Some other studies have sought to improve the accuracy of measurements. Kucukkaya et al. [23] introduced a technique for determining the mounting parameters using computed tomography, and it was especially useful in cases with rotational deformity. Liu et al. [26] accurately measured the deformity and mounting parameters using the elliptic registration technique and three-dimensional reconstruction. Gessmann et al. [31] considered the mounting parameters can be accurately measured by X-ray techniques using calibration markers and a software calibration tool.

The aforementioned techniques are based on precise parameter calculation or the orthogonality of radiographs, none of them focused on the influence of bony ends’ collision on the effectiveness of fracture reduction process. This collision between irregular bony ends often results in an incomplete reduction or failed reduction. In those complex cases, this drawback always results in the need for repeating X-rays exposing the patient to further radiation and making the reduction procedure time-consuming.

In the present study, the three key points reduction trajectory of “distraction-derotation-reduction” was used to resolve this problem. In the whole process, the crucial step is “distraction” to provide sufficient space for the relative movement of the two bony ends. Accurate parameter measurements are very important during the process. In this study, including 57 patients with tibial shaft fractures treated by the HEF, there was not the difference between two groups in the final clinical outcome. Although both groups achieved good average outcomes, the average numbers of repeated X-rays after the first postoperative X-ray and mean durations of deformity correction process in Group II were all less than those in Group I. Furthermore, even though no significant difference was observed between the two goups, there was a trend that the mean external fixation time was shorter in Group II than that in Group I. Repeated reduction may be followed by local soft tissue damage of an acutely injured extremity, obstructing the wound and fracture healing. Therefore, according to our experience, the three key point trajectory of “distraction-derotation-reduction” is recommended due to the less repeated reduction procedures, shorter duration of reduction, and lower potential radiation exposure, especially in complex fractures with irregular bony ends.

Small sample size in a single-center could be considered as a limitation of this study. Compared with the conventional one-step reduction trajectory, this three key point trajectory requires a bit more work of the surgeon but the superiority of this method was approved.

## Conclusion

Compared with the conventional one-step reduction trajectory, there is not the difference in final clinical outcome, but the staged correction trajectory provides less X-ray repeating and shorter duration of the reduction process.

## Data Availability

The datasets analyzed during the current study are available from the corresponding author on reasonable request.

## Abbreviations

HEF: Hexapod external fixator
TSF: Taylor spatial frame
AP: Anteroposterior
